# Mechanisms of Induction of Stimulus-Specific Systemic Responses of Photosynthesis in Wheat Plants

**DOI:** 10.3390/ijms27010401

**Published:** 2025-12-30

**Authors:** Maxim Mudrilov, Maria Ladeynova, Polina Pirogova, Darya Kuznetsova, Sofia Obydennova, Vladimir Vodeneev

**Affiliations:** Department of Biophysics, National Research Lobachevsky State University of Nizhny Novgorod, 23 Gagarin Avenue, Nizhny Novgorod 603022, Russia; mtengri@yandex.ru (M.M.); ladeynova.m@yandex.ru (M.L.); poly.h@mail.ru (P.P.); kuznetsova.dar0@gmail.com (D.K.); son.obydennova24@yandex.ru (S.O.)

**Keywords:** abiotic stress responses, stimulus-specific response, phytohormones, photosynthesis

## Abstract

Systemic photosynthetic responses induced by local stimuli are essential for the formation of systemic acquired acclimation. However, the stimulus-specific features of these responses and the mechanisms that underlie their specificity are still unknown. The aim of this study was to identify the mechanisms of the specificity of photosynthetic responses induced by local heating, burning, and wounding in wheat plants. Photosynthetic responses were multiphasic and included an initial activation of photosynthesis followed by two phases of inactivation, fast and long. The parameters of these responses depended on the type of local stimulus. It has been shown that the activity of Ca^2+^ channels and H^+^-ATPase plays a key role in responses to all types of stimuli. Upon burning and wounding, the fast phase of photosynthetic inactivation is induced mainly by the hydraulic wave, whereas its contribution to heat-induced responses is smaller. The long phase of photosynthetic inactivation is mediated by a decrease in stomatal conductance. In the case of heating, the highest amplitude of the long phase of photosynthetic inactivation and the most substantial increase in the jasmonate levels were observed compared to other local stimuli. Jasmonates probably contribute to the long phase of photosynthetic inactivation through stomatal closure. An increase in jasmonate levels upon heating is probably induced by autopropagating signals. These findings suggest that specificity of responses is provided by different contributions of components to a complex long-distance signal.

## 1. Introduction

Photosynthesis is crucial for plant life, influencing their growth and development. Photosynthetic efficiency significantly affects crop yield and productivity, including the accumulation of biomass and beneficial metabolites [1,2]. It is well known that activity of photosynthesis changes under stress conditions such as high or low light intensity, water deficiency, salinity and heavy metal contamination [1,3,4], which leads to the formation of the systemic acquired acclimation (SAA) [5,6].

Numerous works are currently devoted to studying systemic changes in photosynthetic activity in response to local stimuli. There is evidence that local stimuli induce changes in the activity of photosynthesis in unstimulated distal tissues, including those with stimulus-specific features [7,8,9]. These changes are important for the formation of SAA; however, the specificity of responses to various stimuli has not been fully elucidated. Due to the essential role of photosynthetic responses in the formation of SAA [5], it is necessary to study both the stimulus-specific features of responses and the mechanisms that provide this specificity.

A wide range of long-distance plant signals can potentially transmit information about a local stimulus and induce a specific systemic response [8,10,11]. To date, there are only a few studies demonstrating the formation of specific systemic responses to different local stimuli. Such responses include changes in gene expression [6,12], activity of photosynthesis, and transpiration rates [13,14,15,16]. Stimulus-specific responses have been observed upon exposure to stressors such as wounding [13,16], heating [6,14,15], and burning [14,15], which are typical of natural conditions and experimental studies.

The formation of specific systemic responses in unstimulated tissues will occur only if the long-distance signal carries information about the nature and intensity of the local stimulus. It has been previously demonstrated that the parameters of one type of long-distance signal, the variation potential (VP), depend on the type of stimulus [8,10,17]. VP is a complex electrical signal induced cooperatively by hydraulic and chemical signals [8,11], the ratio of the contributions of which can determine the specificity of its parameters [18]. VP propagation in systemic tissues is associated with changes in the level of signaling messengers, such as Ca^2+^, H^+^ and reactive oxygen species (ROS) [8,11,17,19,20]. These signaling messengers can regulate photosynthetic activity through various mechanisms, including conformational changes in light-harvesting complexes, regulation of the xanthophyll cycle, electron transport chain, Calvin cycle, and changes in transpiration rates [2,21,22]. In addition, some changes in photosynthetic activity may be mediated by other signaling systems, such as hormonal ones [23,24]. Recent work showed the important role of jasmonates (JAs) as candidates for systemic regulation of activity of photosynthesis upon local stimulation [25]. JAs can regulate photosynthesis through stomatal closure [26,27]. In addition, systemic production of JAs is controlled by VP [19], probably through the Ca^2+^-mediated decrease in intracellular pH that accompanies the propagation of VP [28]. It is also possible that other phytohormones are involved in the regulation of photosynthesis [23,24]. However, it is unknown which signals or components of the complex signal provide stimulus-specificity of photosynthetic responses. Thus, the aim of this work is to study the mechanisms of specificity of systemic responses of photosynthesis upon local stimulus exposure.

## 2. Results

### 2.1. Systemic Changes in Activity of Photosynthesis in Response to Different Local Stimuli

The study of the mechanisms of formation of stimulus-specific systemic responses of photosynthesis was performed using inhibitor analysis. Due to the low penetration of inhibitors through the epidermis of wheat leaves, experiments with inhibitors were performed using detached wheat leaves. To evaluate the suitability of the detached leaf model, the parameters of photosynthetic responses in whole wheat plants studied previously (Figure S1) [14] and detached wheat leaves were compared (Figure S2).

**Figure 1:**
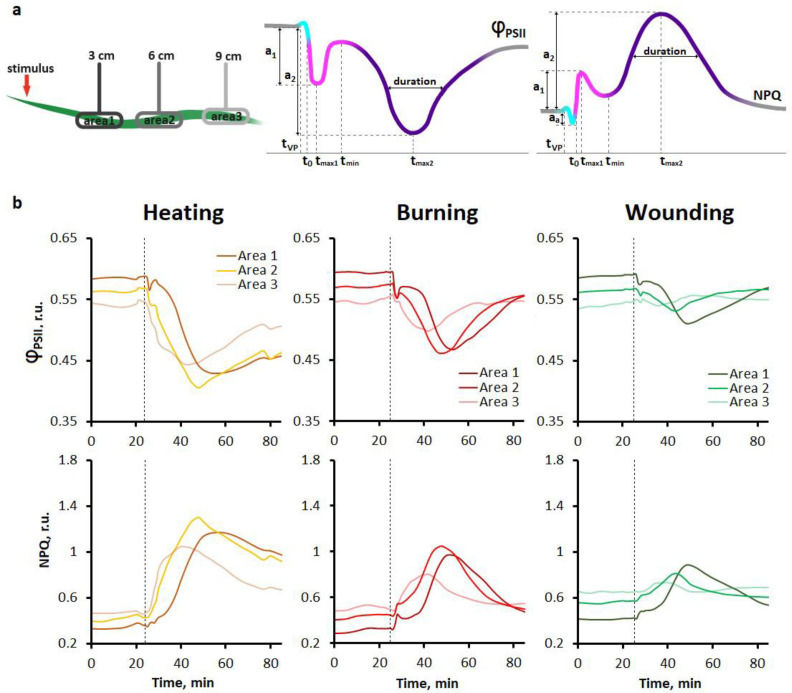
Systemic photosynthetic responses to different local stimuli in detached wheat leaves. (**a**) Schematic representation of the experimental design for monitoring systemic photosynthetic responses in the detached wheat leaf is shown on the left. Schematic representation of the multiphasic photosynthetic response and its parameters is shown on the right. A detailed description is provided in the text. (**b**) Averaged photosynthetic responses induced by local heating, burning or wounding (*n* = 20). The dashed line indicates the moment of generation of the variation potential. Φ_PSII_, effective quantum yield of photochemical reactions of photosystem II; NPQ, non-photochemical fluorescence quenching.

Heating, burning and mechanical wounding caused the generation and propagation of VP in detached leaves (Figures S3 and S4). Note that the attenuation of VP is less pronounced upon heating than upon other local stimuli (Figure S4). VP induced systemic changes in photosynthetic activity, recorded as the spatiotemporal dynamics of the effective quantum yield of photochemical reactions of photosystem II (Φ_PSII_) and non-photochemical fluorescence quenching (NPQ) (Figure 1a). For all types of stimuli, photosynthetic responses were multiphasic and included a short-term transient photosynthetic activation (cyan line) and a biphasic transient photosynthetic inactivation (magenta and purple lines) (Figure 1a). Three parameters were used to characterize these responses: the amplitudes of the first (a_1_) and second (a_2_) phases of photosynthetic inactivation, the slope (the rate of photosynthetic inactivation, a_1_/(t_max1_ − t_0_) and a_2_/(t_max2_ − t_min_)) for the first and second phases, respectively) and the duration (Figure 1a, Table S1). Due to the partial overlap of the first and second phases, the duration of the first phase was calculated as the time interval between the initial decrease in photosynthetic activity (t_0_) and the peak of the first phase (t_max1_). The duration of the second phase was calculated as full width at half maximum. In addition, if possible, the latency for the second phase was calculated as the time interval between the VP generation (t_VP_) in the corresponding area and the initial changes in photosynthetic activity of the second phase (t_min_).

Initial changes in photosynthetic activity, taking into account the measurement frequency of once per minute, were recorded almost simultaneously in all studied areas of the leaf, which may indicate a high speed of propagation of the long-distance signal inducing the systemic photosynthetic response. The short-term photosynthetic activation, corresponding to the increase in Φ_PSII_ and the decrease in NPQ (Figure S5), lasted less than 1.5 min. The biphasic photosynthetic inactivation, corresponding to the decrease in Φ_PSII_ and the increase in NPQ (Figure 1b), was divided into the first fast phase lasting several minutes and the second long phase lasting more than 15 min (Table 1). The long phase began within several minutes after the peak of the fast phase. In some cases, especially in areas more distant from the stimulation zone, a clear division into fast and long phases was not observed. Moreover, the extent of overlap of phases at the same distance varied for individual recordings, as shown in Figure S6. It should be noted that the averaged responses do not fully reflect the multiphasic nature of changes in photosynthetic activity due to differences in the kinetics of responses of individual plants. Individual response curves are shown in Figures S2 and S6.

**Figure 2:**
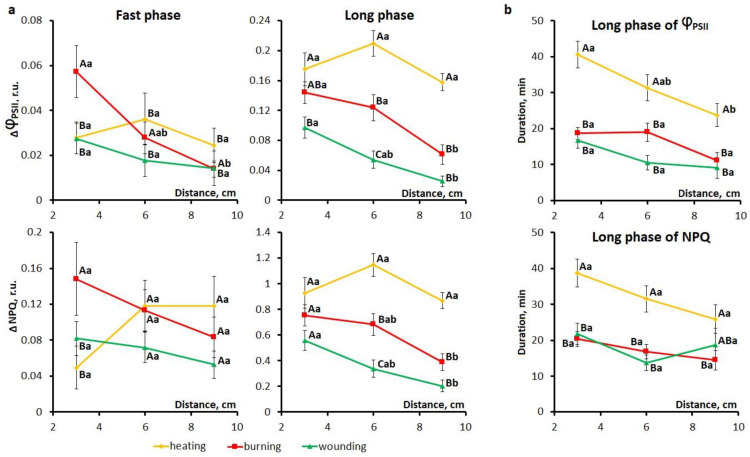
Dependence of the amplitudes of the fast and long phases (**a**) and duration of the long phase (**b**) of photosynthetic inactivation on the distance to the area of local stimulation in detached wheat leaves. Data are represented as Mean ± SEM. Different uppercase letters indicate statistically significant differences between stimuli; different lowercase letters indicate statistically significant differences between distances within a single stimulus (*p* < 0.05). Φ_PSII_, effective quantum yield of photochemical reactions of photosystem II; NPQ, non-photochemical fluorescence quenching.

Differences between local stimuli were observed for the features of the fast and long phases of photosynthetic inactivation (Figure 2a), whereas no differences between stimuli were found for the phase of photosynthetic activation. For the fast phase of photosynthetic inactivation, the highest amplitude was observed near the stimulation site in the case of burning compared to other stimuli. The decrement of the amplitude of the fast phase was greater upon burning than upon wounding. In the case of heating, the decrement of the amplitude of the fast phase was not observed. For the long phase of photosynthetic inactivation, in the case of heating, the amplitude decrement was less pronounced (no statistically significant differences between the distances) (Figure 2a) and duration was the longest compared to other stimuli (Figure 2b), especially in the area near the stimulation site (Table 1). Upon wounding, the amplitude of the long phase of photosynthetic inactivation was the lowest compared to other stimuli and attenuated with distance from the stimulation zone (Figure 2a). Regarding the latency of the second phase, no differences were observed between the stimuli, and the latency was about 5 min and 7 min for Φ_PSII_ and NPQ, respectively (Table 1). Note that the duration of the long phase decreases with increasing distance from the stimulation site (Figure 2b, Table 1).

Comparison of the systemic photosynthetic responses in whole plants (Figure S1) [14] and detached leaves (Figure 1b and Figure 2a) showed their high similarity for all local stimuli used. Nevertheless, some differences were observed between the responses in whole plants and detached leaves, consisting in the magnitude of the amplitude decrement, which is in agreement with those for VP [18]. Thus, the detached leaf model is suitable for studying the mechanisms of formation of stimulus-specific systemic photosynthetic responses induced by VP.

### 2.2. Systemic Changes in Transpiration Rate in Response to Different Local Stimuli

**Figure 3:**
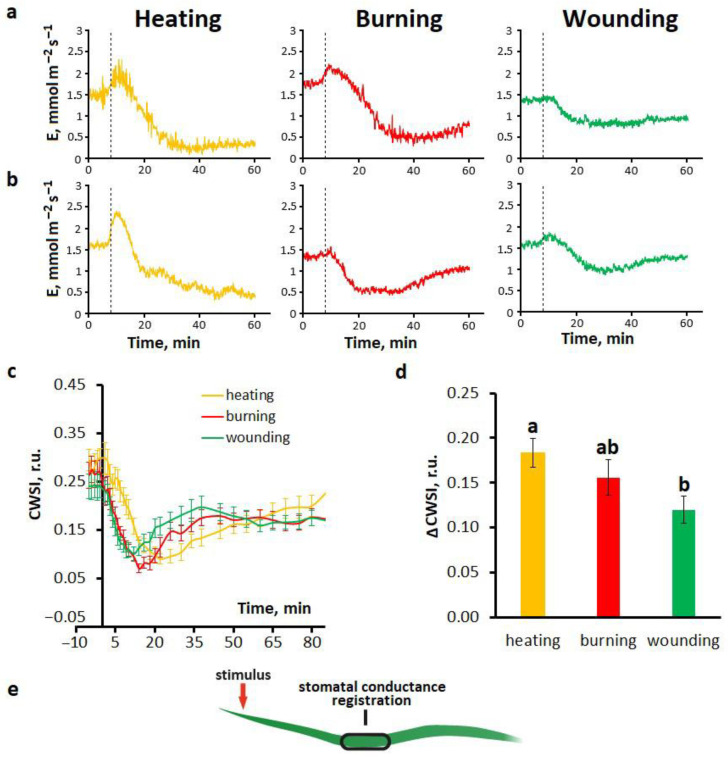
Systemic transpiration responses to local heating, burning or wounding. Representative recordings of stomatal conductance changes in whole wheat plants (**a**) and detached wheat leaves (**b**). The dashed line indicates the moment of generation of the variation potential. Averaged transpiration responses (**c**) and amplitudes of CWSI changes (**d**) in detached wheat leaves. Data are represented as Mean ± SEM (*n* = 10). Different letters indicate statistically significant differences between stimuli (*p* < 0.05). CWSI, crop water stress index. (**e**) Schematic representation of the experimental design for monitoring systemic transpiration responses in the detached wheat leaf.

The observed changes in activity of photosynthesis may be due to changes in stomatal conductance [29]. To characterize systemic transpiration responses to different local stimuli, a qualitative assessment using a gas analyzer and a quantitative analysis using the Crop Water Stress Index (CWSI) were performed. Measurements of the transpiration rate were conducted in the area2 (Figure 1a) due to its significant distance from the stimulation site, but at the same time with high amplitudes of responses and pronounced differences between stimuli. Heating, burning and mechanical wounding of the tip of a wheat leaf caused biphasic changes in the transpiration rate in both whole wheat plants (Figure 3a) and detached wheat leaves (Figure 3b). The first phase was a transient increase in the transpiration rate, lasting about 5–8 min. The second phase was a transient decrease in the transpiration rate, reaching peak levels 15–20 min after the initial decrease. Importantly, a decrease in stomatal conductance was accompanied by the long phase of photosynthetic inactivation, whereas an increase in stomatal conductance was accompanied by both short-term photosynthetic activation and the fast phase of photosynthetic inactivation. It is necessary to note the similarity of the features of systemic transpiration responses in whole plants and detached leaves for all local stimuli used (Figure 3a,b).

Recordings of systemic transpiration responses obtained using thermal imaging as CWSI changes represented a transient decrease in transpiration rate without a preceding increase in transpiration rate (Figure 3c), which may be due to low sensitivity and inertia of this method. Similarly to responses of photosynthesis, transpiration responses were stimulus-specific. The highest amplitude of the decrease in transpiration rate was observed upon heating, and the lowest was observed upon wounding (Figure 3d). Similar differences between stimuli were observed in terms of the duration of transpiration responses (Figure 3b,c).

When comparing the amplitudes of a decrease in stomatal conductance with the amplitudes of the fast and long phases of photosynthetic inactivation, a high correlation was obtained for the long phase, namely 0.87 and 0.86 (*p* < 0,05) for Φ_PSII_ and NPQ, respectively. However, for the fast phase of photosynthetic inactivation, the correlation was lower, about 0.6.

Thus, it was found that both transpiration and photosynthetic responses depend on the type of stimulus, with the main features of the responses being similar for different types of stimuli, especially for the transpiration responses and the long phase of the photosynthetic inactivation. The response of the greatest amplitude and duration is observed upon heating, the least upon wounding, and the intermediate upon burning. The high correlation between amplitudes of transpiration responses and the long phase of photosynthetic inactivation, coupled to the fact that the long phase of photosynthetic inactivation coincided with a decrease in stomatal conductance, suggests that a decrease in the transpiration rate may play a role in the induction of the long phase of photosynthetic inactivation.

### 2.3. Systemic Changes in Hormone Levels in Response to Different Local Stimuli

**Figure 4:**
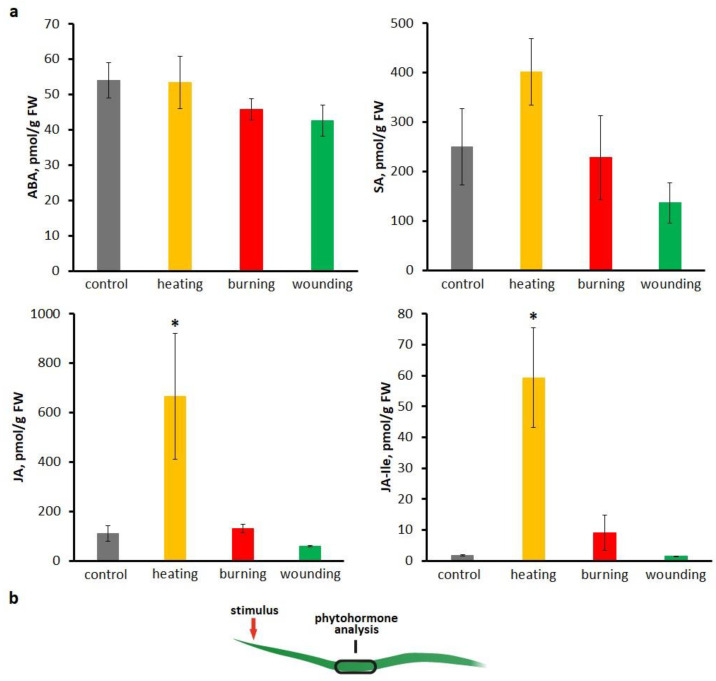
Systemic hormone levels upon different local stimulations. (**a**) Systemic levels of abscisic acid (ABA), salicylic acid (SA), jasmonic acid (JA) and jasmonoyl-isoleucine (JA-Ile) upon local heating, burning or wounding in detached wheat leaves. Data are represented as Mean ± SEM (*n* = 10). * indicates data significantly different from control (*p* < 0.05). (**b**) Schematic representation of the experimental design for quantification of jasmonates in the detached wheat leaf after local stimulation.

Phytohormones are considered as inducers of the systemic photosynthetic responses mediated by the regulation of stomatal conductance [13,25,28,30]. Therefore, quantification of phytohormones in systemic tissues was performed upon exposure to different local stimuli. Phytohormone content was determined in the same area as the transpiration measurements were taken. Levels of abscisic acid (ABA), salicylic acid (SA), jasmonic acid (JA) and its bioactive form, (+)-7-isojasmonoyl-L-isoleucine (JA-Ile), were determined before and 15 min after VP generation, which corresponds to a decrease in transpiration rate and photosynthetic activity during the long phase. No statistically significant changes in ABA and SA concentrations were observed upon exposure to any type of stimulus (Figure 4a). Nevertheless, a tendency for SA levels to increase upon heating was shown. In contrast, JA and JA-Ile levels increased significantly in response to heating. In the case of wounding, levels of JAs remained unchanged, and in the case of burning, a tendency for JA-Ile levels to increase was shown. These findings support a role of JA and JA-Ile in inducing systemic changes in transpiration rate and the long phase of photosynthetic inactivation.

### 2.4. Contribution of H^+^-ATPase to the Systemic Photosynthetic and Transpiration Responses to Different Local Stimuli

Inhibitor analysis of stimulus-specific mechanisms of photosynthetic response formation was performed for one of the chlorophyll fluorescence parameters, namely Φ_PSII_, and in the same area as the transpiration and phytohormone measurements were taken. To study the VP-mediated mechanisms underlying the specificity of the photosynthetic responses, the contribution of H^+^-ATPase inactivation, which plays a key role in VP generation [8,11,20], was analyzed. The mechanisms underlying VP-mediated induction of photosynthetic responses are associated with pH changes occurring during the VP generation [5,31].

**Figure 5:**
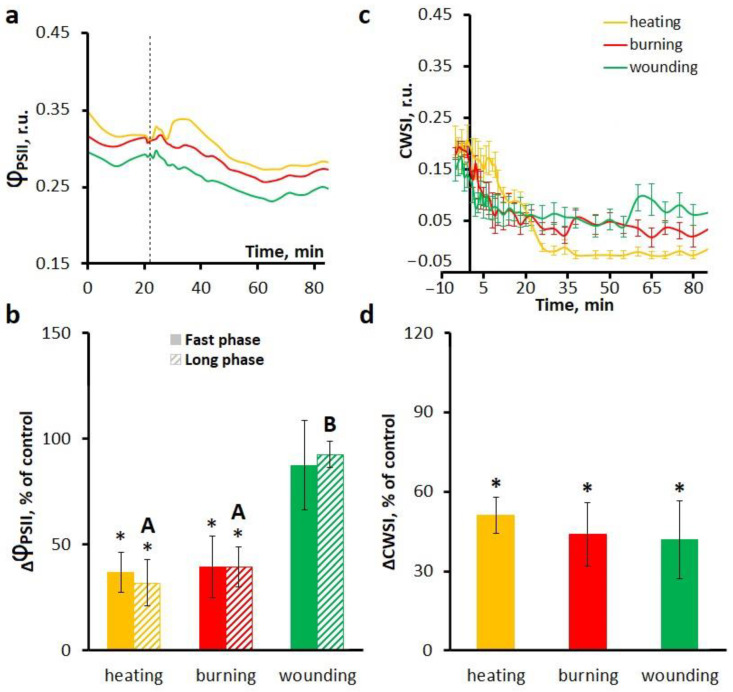
Effects of the H^+^-ATPase inhibitor Na_3_VO_4_ on the systemic photosynthetic and transpiration responses induced by local heating, burning or wounding in detached wheat leaves. Averaged photosynthetic responses (**a**) and amplitudes of fast and long phases of photosynthetic inactivation (**b**). Averaged transpiration responses (**c**) and amplitudes of CWSI changes (**d**). Photosynthetic and transpiration responses were observed at a distance of 6 cm from the stimulation area. The dashed line indicates the moment of generation of the variation potential. Amplitudes are represented as the percentage of control, which is the amplitudes in untreated leaves (without inhibitor). Data are represented as Mean ± SEM (*n* = 10). * indicates data significantly different from untreated leaves (*p* < 0.05). Different letters indicate statistically significant differences between stimuli (*p* < 0.05). Φ_PSII_, effective quantum yield of photochemical reactions of photosystem II; CWSI, crop water stress index.

Treatment with a H^+^-ATPase inhibitor sodium orthovanadate suppressed photosynthesis in unstimulated plants, reducing Φ_PSII_ by about 0.3, and altered systemic photosynthetic responses to local stimuli. Treatment with orthovanadate resulted in an increase in the initial activation of photosynthesis induced by the stimulus. Orthovanadate treatment also reduced the amplitude of the fast phase of photosynthetic inactivation upon heating and burning, but not upon wounding (Figure 5a,b).

Regarding the long phase of photosynthetic inactivation, upon heating and burning, treatment with sodium orthovanadate resulted in an increase in its duration and latency (Table 2), as well as a decrease in its amplitude (Figure 5b). In the case of wounding, similar to the fast phase of photosynthetic inactivation, the amplitude of the long phase remained unchanged (Figure 5b).

Transpiration responses in the presence of sodium orthovanadate were prolonged and reduced in amplitude compared to the control treatment (Figure 5c,d), which is consistent with the effect of orthovanadate on the long phase of decrease in activity of photosynthesis.

### 2.5. Contribution of Ca^2+^ Channels to the Systemic Photosynthetic and Transpiration Responses to Different Local Stimuli

**Figure 6:**
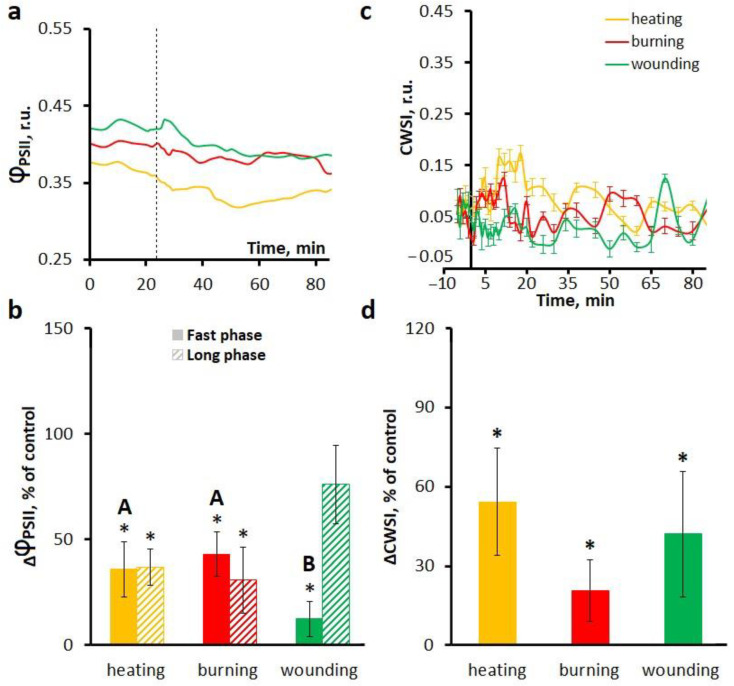
Effects of the Ca^2+^-permeable channel blocker LaCl_3_ on the systemic photosynthetic and transpiration responses induced by local heating, burning or wounding in detached wheat leaves. Averaged photosynthetic responses (**a**) and amplitudes of fast and long phases of photosynthetic inactivation (**b**). Averaged transpiration responses (**c**) and amplitudes of CWSI changes (**d**). Photosynthetic and transpiration responses were observed at a distance of 6 cm from the stimulation area. The dashed line indicates the moment of generation of the variation potential. Amplitudes are represented as the percentage of control, which is the amplitudes in untreated leaves (without inhibitor). Data are represented as Mean ± SEM (*n* = 10). * indicates data significantly different from untreated leaves (*p* < 0.05). Different letters indicate statistically significant differences between stimuli (*p* < 0.05). Φ_PSII_, effective quantum yield of photochemical reactions of photosystem II; CWSI, crop water stress index.

Ca^2+^ is an important regulator of photosynthesis [2]. Similarly to orthovanadate treatment, the treatment with a Ca^2+^-permeable plasma membrane channel blocker LaCl_3_ reduced photosynthetic activity in unstimulated plants. Treatment with Ca^2+^ channel blocker also altered stimulus-induced systemic photosynthetic responses. In addition, treatment with LaCl_3_ reduced the amplitude (Figure 6a,b) and increased the duration (Table 2) of the fast phase of photosynthetic inactivation. Regarding the long phase of photosynthetic inactivation, its amplitude was reduced (except for wounding) (Figure 6b), the duration was extended to about 60 min, and the latency was longer by more than 4–5 min upon LaCl_3_ treatment compared to the control (Table 2).

Transpiration responses in the presence of LaCl_3_ were prolonged and suppressed compared to the control treatment for all local stimuli used (Figure 6c,d).

### 2.6. Contribution of Mechanosensitive Channels to the Systemic Photosynthetic and Transpiration Responses to Different Local Stimuli

**Figure 7:**
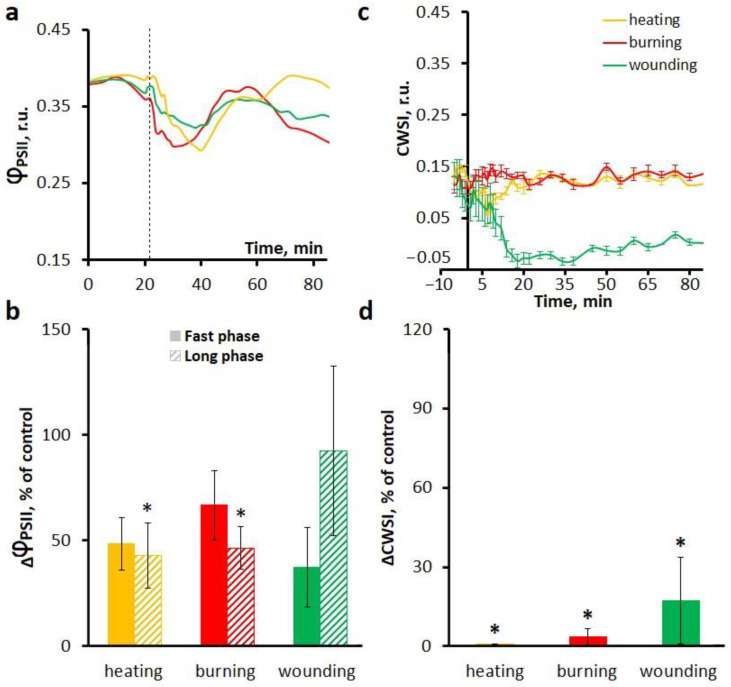
Effects of the mechanosensitive channel inhibitor GdCl_3_ on the systemic photosynthetic and transpiration responses induced by local heating, burning or wounding in detached wheat leaves. Averaged photosynthetic responses (**a**) and amplitudes of fast and long phases of photosynthetic inactivation (**b**). Averaged transpiration responses (**c**) and amplitudes of CWSI changes (**d**). Photosynthetic and transpiration responses were observed at a distance of 6 cm from the stimulation area. The dashed line indicates the moment of generation of the variation potential. Amplitudes are represented as the percentage of control, which is the amplitudes in untreated leaves (without inhibitor). Data are represented as Mean ± SEM (*n* = 10). * indicates data significantly different from untreated leaves (*p* < 0.05). Φ_PSII_, effective quantum yield of photochemical reactions of photosystem II; CWSI, crop water stress index.

To evaluate the involvement of mechanosensitive channels in the induction of the photosynthetic and transpiration responses, leaves were treated with GdCl_3_, an inhibitor of these channels. Similarly to orthovanadate and LaCl_3_ treatments, the treatment with GdCl_3_ reduced photosynthetic activity in unstimulated plants. A strong overlap between the fast and long phases of photosynthetic inactivation was observed in leaves pretreated with GdCl_3_ (Figure 7a,b). In addition, the duration of the long phase of photosynthetic inactivation was shortened upon GdCl_3_ treatment, except in cases of wounding (Table 2). After the long phase of photosynthetic inactivation, irregular fluctuations in the parameters of the light-dependent reactions of photosynthesis were observed without recovery to steady-state levels (Figure 7a,b).

A tendency to a decrease in the amplitude of the fast phase of photosynthetic inactivation upon the treatment with GdCl_3_ was shown (Figure 7a,b). The GdCl_3_-induced suppression of the long phase of photosynthetic inactivation was less pronounced upon wounding than upon burning and heating (Figure 7a,b). In the case of wounding, this decrease in amplitude was statistically insignificant, while the amplitude of ΦPSII was reduced to 46% upon burning and to 43% upon heating.

Transpiration responses in the presence of GdCl_3_ were suppressed to a lesser extent upon wounding, whereas transpiration responses induced by heating and burning were suppressed almost completely (Figure 7c,d).

### 2.7. Contribution of Reactive Oxygen Species to the Systemic Photosynthetic and Transpiration Responses to Different Local Stimuli

**Figure 8:**
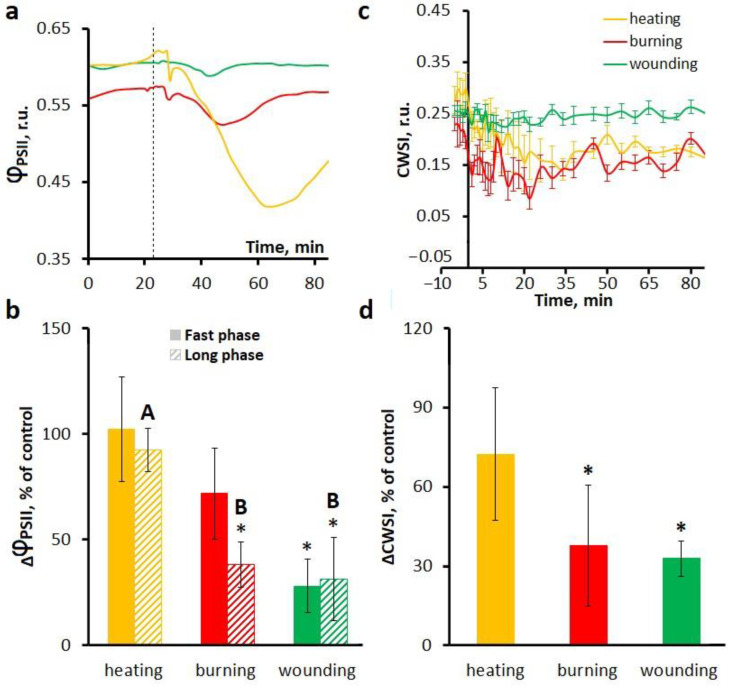
Effects of the ROS scavenger N,N′-dimethylthiourea (DMTU) on the systemic photosynthetic and transpiration responses induced by local heating, burning or wounding in detached wheat leaves. Averaged photosynthetic responses (**a**) and amplitudes of fast and long phases of photosynthetic inactivation (**b**). Averaged transpiration responses (**c**) and amplitudes of CWSI changes (**d**). Photosynthetic and transpiration responses were observed at a distance of 6 cm from the stimulation area. The dashed line indicates the moment of generation of the variation potential. Amplitudes are represented as the percentage of control, which is the amplitudes in untreated leaves (without inhibitor). Data are represented as Mean ± SEM (*n* = 10). * indicates data significantly different from untreated leaves (*p* < 0.05). Different letters indicate statistically significant differences between stimuli (*p* < 0.05). Φ_PSII_, effective quantum yield of photochemical reactions of photosystem II; CWSI, crop water stress index.

ROS waves can be involved in the induction of systemic photosynthetic responses [32,33,34,35]. Treatment with a ROS scavenger N,N′-dimethylthiourea (DMTU) did not change photosynthetic activity in unstimulated plants. The shape of the stimulus-induced photosynthetic responses also did not change in leaves pretreated with DMTU (Figure 8a,c).

Treatment with DMTU had no effect on the heat-induced photosynthetic responses, but reduced the amplitude and duration of the fast and long phases of photosynthetic inactivation upon burning and wounding, with the exception of the fast phase upon burning, for which the reduction was statistically insignificant (Figure 8a,b).

Treatment with DMTU did not cause significant changes in the dynamics of transpiration responses (Figure 8c). However, the amplitude of transpiration responses in the presence of DMTU was reduced upon wounding and burning, but statistically insignificantly upon heating (Figure 8d), which is consistent with the effect of DMTU on the long phase of inactivation of photosynthesis.

## 3. Discussion

### 3.1. Stimulus-Specific Features of Systemic Photosynthetic Responses

Studying the parameters of systemic photosynthetic responses induced by heating, burning, and wounding revealed both general and stimulus-specific features. For all local stimuli used, photosynthetic responses were multiphasic and included a short-term initial activation of photosynthesis with a pronounced decrement and a biphasic inactivation of photosynthesis, divided into fast and long phases (Figure 1). In the case of heating, the long phase of inactivation of photosynthesis was high-amplitude and without a pronounced decrement, whereas in the case of wounding, photosynthetic responses were low-amplitude with a decrement for both phases of inactivation. In the case of burning, a high amplitude of the fast phase of inactivation near the stimulation site and a pronounced decrement were observed (Figure 2). In summary, wounding and burning induced responses that attenuated with distance, whereas the attenuation was less pronounced upon heating.

The revealed dependence of parameters of systemic responses on the type of local stimulus is consistent with results from other studies. The dependence of parameters of systemic responses on the type of stimulus, such as wounding [13,16,36,37,38], heating [6,14,15,37,38], burning [14,15] and high light [36,38,39], has been show for changes in gene expression [6,36], photosynthesis and transpiration [13,14,15,16]. However, the mechanisms underlying stimulus-specific differences in features of systemic responses remain unknown.

Photosynthetic responses were detected in unstimulated distal tissues, which means that to induce specific response, information about the nature of the stimulus must be transmitted by long-distance signals. Local stimuli cause the simultaneous induction of a number of long-distance signals, such as electrical, hydraulic and chemical, including autopropagating Ca^2+^ and ROS waves [10,11,17,20,40]. The propagation of these signals induces a complex of changes in systemic tissues, including changes in the levels of key signaling messengers such as Ca^2+^, H^+^, and ROS [8,10,11,19,31,40], which may underlie the formation of systemic responses, including photosynthetic responses [8,9,31].

### 3.2. Mechanisms of Induction of Systemic Photosynthetic Responses

The study of stimulus-specificity of responses requires the identification of general mechanisms of their formation. Numerous works have been devoted to the study of complex mechanisms regulating photosynthesis [1,2,21,41,42,43]. One of the most important regulators of photosynthetic processes are Ca^2+^ [2,21], the change in the level of which is associated with the propagation of long-distance signals [8,11,19]. Ca^2+^-dependent regulation of photosynthesis has been shown both at the level of gene expression, such as the xanthophyll cycle regulator protein gene *VDE* (*violaxanthin de-epoxidase*) [2], and at the post-translational level [2,21]. Direct Ca^2+^-dependent or calmodulin-mediated regulatory pathways have been demonstrated for photosystem assembly through D1 and PsbS proteins (the subunits of photosystem 2); the electron transport chain for PsaN (the subunits of photosystem 1) and NAD kinase 2 (NADK2) proteins; antioxidant systems including ascorbate peroxidase, superoxide dismutase, and catalase; dark stages of photosynthesis through the Calvin cycle, transketolase, fructose-l,6-bisphosphatase/sedoheptulose-1,7-bisphosphatase (FBPase/SBPase), and 12 kDa chloroplast protein (CP12); and much more [2,44,45,46].

In addition to the direct effect on the activity of light-dependent reactions, Ca^2+^ can mediate changes in pH by regulating the activity of a key enzyme, H^+^-ATPase [8,20]. Changes in pH, in turn, can lead to changes in many photosynthetic processes, such as changes in the conformation of light-harvesting complexes [1,41,43,47,48,49], regulation of the Calvin-Benson cycle both by changing the activity of its enzymes, such as FBPase/SBPase [2,49], and by regulating CO_2_ availability for chloroplasts by changing the CO_2_:HCO_3_^-^ ratio or activity of aquaporins [5,31,42].

An important mechanism for changing the photosynthetic activity is the regulation of stomatal conductance, which depends on the activity of Ca^2+^-channels and H^+^-ATPase, as well as on ROS levels [50,51,52,53] and, most importantly, on various phytohormones such as ABA, SA and JAs [52,53]. It is well known that ABA plays a key role in the closure of stomata [54]. However, no changes in ABA levels were observed 15 min after local stimulation (Figure 4), which is consistent with data on a later peak of ABA concentration compared to JA and JA-Ile in systemic tissue [55]. Whereas an increase in levels of JAs preceded a decrease in stomatal conduction, supporting a role of JAs in stomatal closure in systemic tissues upon local stimulation [25].

### 3.3. The Multiphasic Nature of Systemic Photosynthetic Responses and Potential Inducers of Each Phase

The multiphasic nature of systemic photosynthetic responses suggests that individual phases of the response are induced by different long-distance signals or different components of a complex long-distance signal. However, which specific signals or their components trigger the stimulus-specific formation of each of the identified phases is unknown.

**Figure 9:**
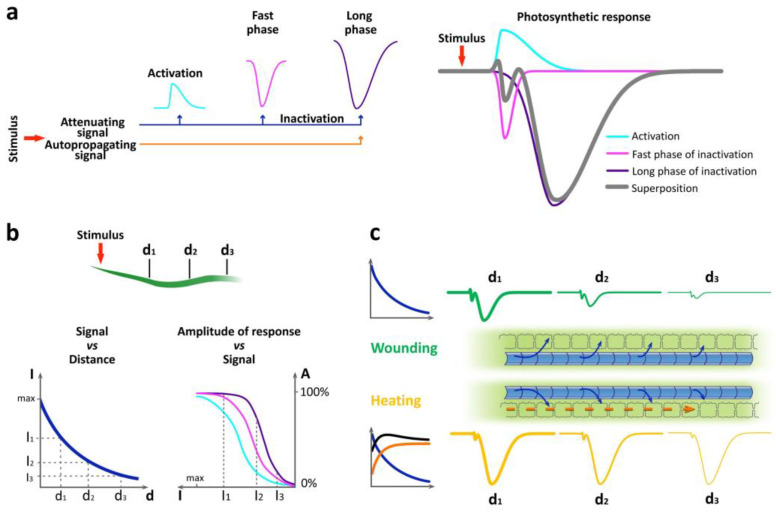
Hypothetical model for the formation of a multiphasic photosynthetic response in systemic tissues and its spatial dynamics induced by different local stimuli. (**a**) Scheme of formation of a multiphasic photosynthetic response as a superposition of individual components induced by long-distance signals. The components of the response are presented as curves corresponding to the initial activation (cyan), fast (magenta) and long (purple) phases of inactivation. Superposition of putative components represents changes in effective quantum yield of photochemical reactions of photosystem II. (**b**) Scheme showing the dependence of the amplitudes of the signal and the phases of the photosynthetic response on distance. The dependence of the intensity of the signal attenuating with distance is shown on the left. An exponential dependence of signal intensity on distance is assumed. Dependences of the amplitudes of each response component on the signal intensity for different distances from the stimulation area are shown on the right. A typical sigmoid dependence of the response amplitude on the signal intensity is assumed. (**c**) Schematic representation of the features of the spatial dynamics of the photosynthetic response induced by different stimuli, using wounding and heating as examples. A schematic representation of the dependence of the intensity of wound- and heat-induced signals on the distance from the stimulation site is shown on the left. In the case of wounding, only an attenuating signal (blue) appears to propagate, whereas in the case of heating, an additional autopropagating signal (orange) is transmitted. Key differences between photosynthetic responses induced by different local stimuli are the ratio of response phases and spatial dynamics of the response, which are determined by differences in long-distance signals. Blue arrows indicate an attenuating signal transmitted through the vessels from the stimulation zone. The orange dashed arrow indicates an autopropagating signal transmitted through the symplastic pathway. d, distance; I, intensity.

To describe the putative mechanisms that determine the identified features of systemic photosynthetic responses, a modeling approach was used, namely, decomposition of the response into three different components: the initial activation of photosynthesis (I), the fast phase of inactivation of photosynthesis (II), and the long phase of inactivation of photosynthesis (III) (Figure 9a). Plants are known to have both attenuating signals, such as chemical and hydraulic ones [56,57], and autopropagating ones, which propagate over long distances without significant attenuation [33,34,58]. The signal intensity in systemic tissues depends on the stimulus strength, which determines the initial amplitude, and the distance from the stimulation area [7,8,59]. In turn, the parameters of each phase of the photosynthetic response, such as amplitude and duration, can have individual dependencies on the signal intensity, differing in threshold and slope (Figure 9b). The identified features of photosynthetic responses (Figure 2, Table S1) support this hypothesis. Hypothetical curves of wound-induced and heat-induced photosynthetic responses, which represent a superposition of the putative components, are shown for different distances from the stimulation area (Figure 9c).

The initial activation of photosynthesis may be due to hydropassive opening of stomata [14,60], which is supported by the fact that the increase in transpiration rate coincides with the initial photosynthetic activation.

The almost simultaneous beginning of the fast phase of photosynthetic inactivation was observed at different distances from the stimulation zone. This phase of the response exhibited a substantial decrease in amplitude with distance upon burning and wounding, which appears to be determined by the attenuating signal that induced this response (Figure 9). Attenuation with distance is characteristic of both the hydraulic signal and the diffusion of wound substance from the stimulation zone [10,56,57,59]. Identified high propagation speed of the inducing signal, at least 9 cm/min, indicates a hydraulic signal [10,57].

In the case of burning, the high amplitude of the fast phase of photosynthetic inactivation near the stimulation site may be due to a stronger hydraulic signal, as evidenced by previous data [18]. The hydraulic signal may determine the amplitude of the response through the level of Ca^2+^ increase, of which a concentration-dependent increase has been shown under osmotic stress [61,62].

The induction of the fast phase of photosynthetic inactivation can be mediated by Ca^2+^ influx through mechanosensitive channels directly or indirectly through activation of other Ca^2+^ channels, such as the glutamate receptor-like proteins (GLRs), mediated by the anion stretch-activated MscS (mechanosensitive channel of small conductance)-like (MSL) 10 [63]. As noted above, Ca^2+^ can regulate responses at both transcriptional and post-translational levels, but the absence of a significant lag period between stimulation and the beginning of the fast phase of photosynthetic inactivation suggests a post-translational level. The post-translational pathway may involve the influence of Ca^2+^ on light-dependent reactions, or through limitation of transpiration. However, during the fast inactivation phase of photosynthesis, an increase in the transpiration rate is observed, which rules out this pathway. Thus, pathways of Ca^2+^ influence on light-dependent reactions will play a role. Since the fast phase of inactivation of photosynthesis is short-term and transient, the mechanism of its formation appears to be Ca^2+^-mediated inactivation of the H^+^-ATPase and a subsequent decrease in pH [14], followed by protonation of the PsbS protein and reversible conformational changes in the light-harvesting complex II (LHCII) [41,43,48,49,64]. Direct effects of Ca^2+^ on the activity of light-dependent reactions described above cannot be ruled out.

We assume that the processes leading to an increase and decrease in photosynthetic activity partially compensate each other, that is, the summation of the corresponding curves leads to a resulting response with smaller phase amplitudes than they could be individually. This is well illustrated by superimposing curves (Figure 9a). It is possible that the small amplitude of the fast phase of photosynthetic inactivation upon heating compared to burning may also be associated with a compensatory effect (Figure S7) of increasing the transpiration rate (Figure 3).

The presence or absence of a clearly visible boundary between the extremes of the integral curve of the photosynthetic response may be due to the different extent of overlap of individual phases. Based on our assumptions, there are different dependencies of the amplitudes of each phase of the response on the signal intensity (Figure 9b). Thus, the disappearance of a clearly visible boundary between the first and second phases of inactivation, which occurs with increasing distance from the stimulation zone, can be caused by a decrease in the amplitude of the initial activation with distance (Figure S7).

Similarly, the decrease in the latency of the long phase with increasing distance may only be apparent. Near the stimulation site, the high amplitude of the fast phase may mask the onset of the formation of the long phase. The rapid attenuation of the amplitude of the first phase with distance allows us to see the true time of the beginning of the second phase.

The high correlation between the amplitudes of transpiration responses and the long phase, coupled with their contemporaneity, supports the hypothesis [14] that stomatal closure plays a role in the induction of the long phase of inactivation of photosynthesis by reducing the availability of CO_2_ [5,31,42]. It was noted above that Ca^2+^ can cause the closure of stomata [52,53], but this process is not instantaneous, as is the decrease in internal CO_2_ concentration caused by the closure of stomata.

Superposition of the hypothetical curves showed that in the case of the attenuating long phase of photosynthetic inactivation, the initial inducer of changes could be the same signal, but with some delay, potentially caused by the above mentioned processes. However, in the case of heating, the non-attenuating long phase requires the propagation of an additional signal, providing a higher amplitude and duration compared to other stimuli even at a long distance from the stimulation site. This also indicates an additional inducer of the long phase of photosynthetic inactivation induced by heating. The small decrement in the amplitude, which was observed both in detached leaves in this work and in whole plants in the previous study [14], supports the induction of this phase by an autopropagating signal. The presence of autopropagating waves of ROS and Ca^2+^ in plants has been demonstrated previously [33,34,58,65,66].

There is a question about the absence of such an additional autopropagating signal in the case of other stimuli studied, including such strong ones as a burning. We assume that upon burning and wounding, the transmission of an autopropagating signal is prevented by disruptions in the hydraulic continuum caused by the closure of plasmodesmata, which, in turn, are caused by changes in pressure in cells due to their destruction by these damaging stimuli [67,68]. The importance of the symplastic pathway for autopropagating waves has been previously shown [69]. It was shown in wheat [14,18] and pea [15] plants that the VP did not attenuate in the case of heating and attenuated in the case of burning [14,15,18] and wounding [15,18]. It is known that the parameters of signal waves can correlate with each other; thus, the suppression of Ca^2+^ and electrical waves are often observed together [70,71]. Taken together, these evidence suggest that autopropagating waves are transmitted only in response to heating, whereas their absence upon burning and wounding may be due to the disruptions in the symplastic pathway.

Based on the above, a suitable candidate for the element of the autopropagating signal generation system is the vacuolar cation channel TWO PORE CHANNEL 1 (TPC1) [11], which is activated by simultaneous depolarization of the vacuolar membrane and an increase in cytosolic Ca^2+^ concentrations. Moreover, depolarization of the vacuolar membrane can be caused by Ca^2+^-activated K^+^-selective channels two pore potassium (TPK) of vacuoles upon the exposure to various stimuli [72,73]. Furthermore, TPC1 activation appears to be stimulus-specific, as TPC1 is not involved in the increase in cytosolic Ca^2+^ concentrations in response to cold, hyperosmotic stress, salt stress, and the biotic markers translation elongation factor Tu (elf18) and fragment of bacterial flagellin (flg22) [74]. However, there is conflicting data on the involvement of TPC1 in the formation of the systemic Ca^2+^ wave upon wounding in arabidopsis [56,75,76]. The presence of evidence for the involvement of autopropagating waves in responses to wounding may serve as a refutation of the proposed hypothesis, but species-specific features of response induction upon the exposure to various stimuli cannot be ruled out.

Autopropagating Ca^2+^ signal can induce transpiration suppression through the production of phytohormones, primarily JAs [55]. It has been previously shown that Ca^2+^ signal-mediated pH changes induced the conversion of the 12-oxo-phytodienoic acid to JA and JA-Ile [28], with a subsequent decrease in stomatal conductance and the induction of long-term suppression of photosynthesis [25]. In this study, heating caused the highest increase in JA and JA-Ile levels, whereas in the case of burning, there was only a tendency to increase, and in the case of wounding, the levels did not increase, supporting our hypothesis. Furthermore, TPC1 has been shown to be involved in JA synthesis [72,77] and Ca^2+^-induced stomatal closure [72,78].

### 3.4. Effects of Inhibitors on Photosynthetic Responses

The findings of the inhibitor analysis are consistent with the model of the formation of a stimulus-specific photosynthetic response. Inhibitor analysis showed that Ca^2+^ channels and H^+^-ATPase are required for the formation photosynthetic and transpiration responses to all types of stimuli, suggesting a role for Ca^2+^ in H^+^-ATPase inactivation, which is consistent with previous results for VP [18]. The fact that complete suppression of responses is not observed may be due to non-specific Ca^2+^ influx through other channels that may have a predominantly non-calcium permeability. It should be noted that the inhibitors used, such as Na_3_VO_4_ and LaCL_3_, suppressed the activity of photosynthesis before stimulation (in unstimulated plants), which was also shown in previous studies [79,80].

Regarding the long phase of photosynthetic inactivation, the shift in the peak of stimulus-induced JA and JA-Ile dynamics in LaCL_3_-treated plants [28] corresponds to the shift in the onset of the long phase upon the same LaCL_3_ treatment in this work (Table 2). The less pronounced effect of LaCL_3_ treatment on the long phase of photosynthetic inactivation upon wounding indicates the involvement of other induction pathways not inhibited by Na_3_VO_4_ and LaCL_3_.

Treatment with DMTU, a ROS scavenger, affected responses induced by burning or wounding, but not responses caused by heating. This may be due to the different contribution of ROS propagating from the stimulation zone due to cell damage [81] to the induction of photosynthetic and transpiration responses, which was demonstrated by differences in the amplitude of the increase in ROS levels with different stimuli [35]. It is known that ROS may be involved in stomatal closure [50,51]. In cases of burning and wounding, the contribution of ROS propagating from the damaged area to the decrease in stomatal conductance was probably higher than upon heating.

Treatment with GdCl_3_ resulted in similar effects for all stimuli. It can be assumed that the activation of mechanosensitive channels is required to induce the fast phase of decrease in photosynthetic activity upon stimulation with any type of stimulus. Such channels may be MSL10 [63] whose activity is inhibited by GdCl_3_ [82]. In the case of wounding, GdCl_3_ treatment did not result in suppression of the long phase of photosynthetic inactivation, suggesting a smaller contribution of mechanosensitive channels to its induction upon wounding. In the case of heating, it cannot be ruled out that mechanosensitive channels are involved in autopropagating Ca^2+^ waves and subsequent production of phytohormones. Moreover, this mechanism is consistent with the squeeze cell hypothesis to trigger JA biosynthesis [11,83].

### 3.5. Mechanisms of Formation of Stimulus-Specific Systemic Responses of Photosynthesis

**Figure 10:**
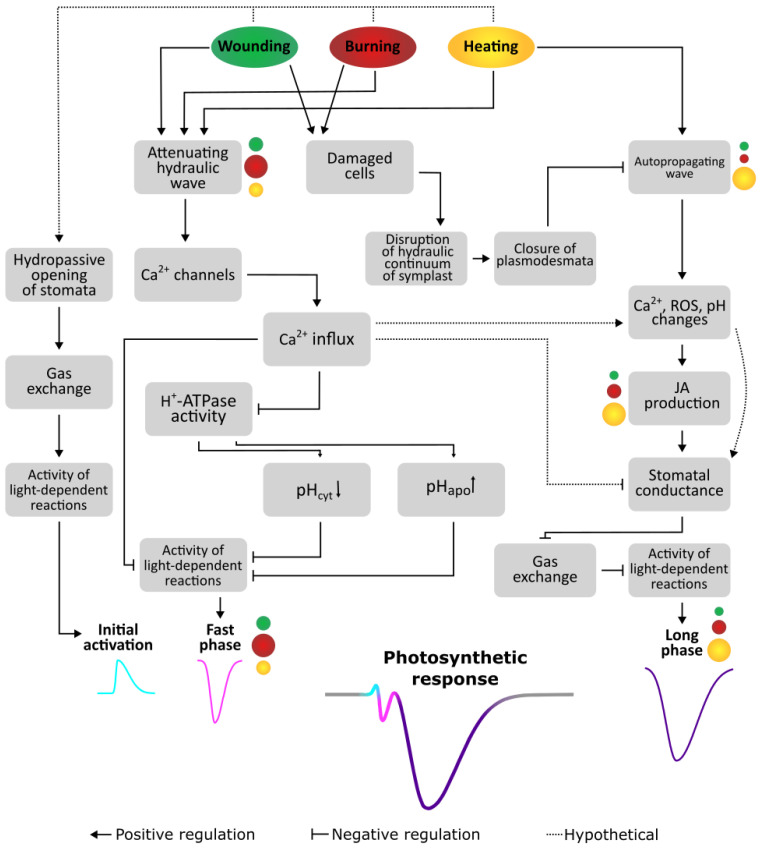
Model for the formation of systemic photosynthetic responses induced by different local stimuli. The color of the circle indicates the type of stimulus. The wounding is shown in green, the burning in red and the heating in yellow. The size of the circle indicates the relative amplitude of the corresponding changes for different stimuli. A detailed description of the sequence of events presented in the model is provided in the text.

Based on the analysis of the obtained results, we proposed the mechanism for the formation of systemic stimulus-specific responses of photosynthesis (Figure 10). Local stimulation induces an attenuating hydraulic wave and the propagation of wound substance transported by xylem mass flow. In case of a burning, a strong hydraulic wave is observed, whereas in cases of wounding and heating, it is reduced. In addition, in the cases of burning and wounding, direct destruction of cells is observed, leading to the transmission of pressure waves through the symplastic pathway, causing the closure of plasmodesmata, whereas in the case of heating this is not observed. The hydraulic wave causes hydropassive opening of the stomata, which, on the one hand, leads to an increase in the rate of transpiration, and on the other hand, to an initial photosynthetic activation. Hydraulic mass flow causes activation of mechanosensitive and ligand-gated Ca^2+^-permeable channels, which leads to Ca^2+^ influx, inducing a decrease in the activity of light-dependent reactions, forming the fast phase of photosynthetic inactivation. The parameters of the fast phase of photosynthetic inactivation correlate with the parameters of VP, which is due to the dependence of the parameters of both on pH changes. The propagation of attenuating ROS waves from the damaged area initiates the closure of stomata upon burning and wounding. Stomatal closure results in proportional reductions in CO_2_ availability and photosynthetic activity during the long phase. In the case of heating, the autopropagating signal induces production of JAs in systemic tissues and further enhances the reduction in transpiration rate.

In general, the revealed differences in photosynthetic responses between stimuli are mainly quantitative rather than qualitative, and there are unidirectional changes of varying amplitude and duration. Nevertheless, such quantitative changes may result in qualitatively different final defense responses. It is known that changes in the amplitude of signals and the functional responses they induce can lead to an inversion of resistance to subsequent stressors [84].

## 4. Materials and Methods

### 4.1. Plant Material and Growth Conditions

Wheat (*Triticum aestivum* L.) cv. Daria was grown at 23 °C during 16 h of light and 8 h of dark. For all experiments, 15–21-day-old plants, grown in pots with sand, were used. Due to the low penetration of chemicals through the epidermis of wheat leaves, experiments with inhibitors were performed using detached wheat leaves (second mature leaf 17 cm long) cut from plants and incubated in a standard solution (1 mM KCl, 0.5 mM CaCl_2_, 0.1 mM NaCl) for 17 h.

### 4.2. Local Stimulation

Local stimuli were applied to the tip of the leaf, 1 cm long. Three types of local stimuli were used: (1) gradual heating in a water-filled cuvette to 60 °C for 6–7 min; (2) burning with a flame for 3 s; (3) mechanical wounding by crushing with a plastic cylinder. The selection of stimuli is determined by the need to compare the obtained results with published data [7,8,10,31]. A single stimulation experiment was carried out for individual plant. Prior to experiments, plants were moved from the growth room and acclimated for 1 h in the recording room at 23 °C.

### 4.3. Extracellular Recordings of Electrical Signals

Surface potential changes were recorded using Ag^+^/AgCl macroelectrodes EVL-1M3 (Gomel Plant of Measuring Devices, Gomel, Belarus) filled with 3 M KCl. The macroelectrodes were connected to a high-impedance amplifier IPL-113 (Semico, Novosibirsk, Russia). Three measuring macroelectrodes were placed on the leaf with an inter-electrode distance of 3 cm. The distance between the stimulation zone and the first electrode was also 3 cm. Measuring macroelectrodes were connected with leaves by cotton threads moistened with a standard solution. A reference electrode was placed in a standard solution surrounding the leaf cut. Electrical potential recordings were acquired at 1 Hz.

### 4.4. Photosynthetic Activity Measurements

Pulse-Amplitude-Modulation (PAM) fluorometry was used to detect changes in the activity of photosynthesis. PAM imaging was carried out using Open FluorCam FC 800-O/1010 PAM-fluorimeter (Photon Systems Instruments, Drásov, Czech Republic). Changes in photosynthetic activity were assessed by the spatiotemporal dynamics of two parameters, effective quantum yield of photochemical reactions of photosystem II (Φ_PSII_) and non-photochemical fluorescence quenching (NPQ).

Wheat leaves were placed in the measurement system and adapted to the experimental conditions for 60 min. F_0_ and F_m_ were determined after 15 min of dark adaptation, after which actinic light illumination (280 μmol m^−2^ s^−1^, 617 nm) was turned on for 15 min. Saturating pulses (4000 μmol m^−2^ s^−1^, cool white light, 6500 K) were then turned on.

### 4.5. Transpiration Rate Measurements

A GFS-3000 gas analyzer (Heinz Walz GmbH, Effeltrich, Germany) and Dual-PAM gas-exchange Cuvette 3010-Dual common measuring head (Heinz Walz GmbH, Effeltrich, Germany) were used for measurements of stomatal conductance, which was automatically calculated by GFS-Win software (version 3.82) (Heinz Walz GmbH, Effeltrich, Germany). The conditions in the measuring cuvette were as follows: blue actinic light 460 nm and 240 µmol m^−2^ s^−1^, temperature 23 °C, relative humidity about 70%, CO_2_ concentration 360 ppm. The measuring cuvette was placed on the leaf at a distance of 6 cm from the stimulation site. Transpiration rate recordings were acquired at 1 Hz.

Leaf temperature was measured using a thermal imager Testo 885 (Testo, Lenzkirch, Germany). Infrared images were analyzed using IRSoft software (Testo, Lenzkirch, Germany). The leaf surface temperature (T), the temperatures of dry (T_dry_) and moisture standards (T_moisture_) were measured before stimulation and within 120 min after stimulation. Changes in transpiration rate were assessed using the crop water stress index (CWSI): CWSI = (T_dry_ − T)/(T_dry_ − T_moisture_).

### 4.6. Quantification of Hormones

The levels of ABA, SA, JA, and JA-Ile were determined in a 5 cm long fragment of the wheat leaf, which was located at a distance of 5 cm from the stimulation site. Leaf fragments were harvested before and 15 min after VP generation and immediately frozen in liquid nitrogen, weighed, and ground to a fine powder. Each sample consisted of five leaf fragments from five plants.

The extraction procedure of the powdered leaf fragments and the quantification of hormone levels were performed as described by Ladeynova et al. (2024) [28]. Hormone levels were measured using an electrospray ionization triple quadrupole tandem mass spectrometer LCMS-8040 (Shimadzu, Kyoto, Japan). The VP generation was monitored using macroelectrodes placed at a distance of 5 cm from the stimulation site, simultaneously with the quantification of hormones.

### 4.7. Inhibitor Studies

To investigate the mechanisms of specificity of systemic photosynthetic responses upon local stimulation, inhibitor analysis was performed using the plasma membrane H^+^-ATPase inhibitor Na_3_VO_4_ (2 mM), the plasma membrane Ca^2+^-permeable channel blocker LaCL_3_ (5 mM), the mechanosensitive channel inhibitor GdCL_3_ (5 mM), the scavenger of ROS DMTU (1 mM). All inhibitors were from Sigma-Aldrich (St. Louis, MO, USA). Solutions for inhibitor treatments were prepared in a standard solution. The solutions were applied to detached wheat leaves using vacuum infiltration. To do this, the cut edge of the detached leaf was submerged in a solution, and then subjected to a single cycle of vacuum infiltration for 5 min at 70 kPa in a vacuum desiccator. The control experiments were conducted in the same manner via infiltration, using a standard solution for infiltration. All experiments were performed 90 min after infiltration.

### 4.8. Statistical Analysis

All experiments involving each type of stimulus were repeated at least ten independent biological replicates, each conducted on a separate plant. The results are presented as means ± SEM, first-order derivative, representative records of individual measurements. The statistical significance of pairwise comparisons was determined using Student’s *t*-test. For multiple comparisons, two-way ANOVA were performed. The level of statistical significance was set at *p* < 0.05. The statistical analysis was performed using MS Excel (Microsoft Corporation, Redmond, WA, USA) and GraphPad Prism software (v. 6.07, GraphPad Software Inc., San Diego, CA, USA).

## 5. Conclusions

This study identified mechanisms of the formation of specific systemic photosynthetic responses upon exposure to different local stimuli. However, further research is needed to understand the full picture, and this research should focus primarily on

- The mechanisms of generation and transmission of various long-distance plant signals;
- The analysis of the relationship between the parameters of a complex long-distance signal and the nature and strength of the stimulus;
- The molecular mechanisms of long-distance signal induction of responses in systemic tissues.

## Figures and Tables

**Table 1:** Duration and latency of the fast and long phases of photosynthetic inactivation for Φ_PSII_ and NPQ upon different local stimulations in detached wheat leaves.

Stimulus	Distance, cm	Duration of the Fast Phase, min		Duration of the Long Phase, min		Latency of the Long Phase, min	
		Φ_PSII_	NPQ	Φ_PSII_	NPQ	Φ_PSII_	NPQ
heating	3	1.6 ± 0.2	2.1 ± 0.3	40.6 ± 3.7 ^a^	38.8 ± 3.9 ^a^	5.8 ± 0.8	7.3 ± 0.6 ^ab^
	6	1.4 ± 0.2	1.7 ± 0.2	31.3 ± 3.6 ^a^	31.5 ± 3.7 ^a^	4.8 ± 0.7	5.6 ± 0.7 ^a^
	9	1.9 ± 0.3	2.6 ± 0.5	25.0 ± 3.1 ^a^	25.6 ± 4.1 ^a^	5.3 ± 1.6	5.0 ± 0.8 ^a^
burning	3	1.8 ± 0.3	2.6 ± 0.4	18.7 ± 1.7 ^b^	20.5 ± 2.2 ^b^	6.0 ± 0.7	8.1 ± 0.5 ^a^
	6	1.6 ± 0.2	2.3 ± 0.5	19.2 ± 2.6 ^b^	17.0 ± 2.1 ^b^	4.2 ± 0.5	5.9 ± 0.4 ^a^
	9	1.4 ± 0.1	2.0 ± 0.2	11.1 ± 2.2 ^b^	14.5 ± 2.8 ^b^	4.9 ± 1.1	6.0 ± 0.6 ^a^
wounding	3	1.7 ± 0.2	2.4 ± 0.3	16.7 ± 2.2 ^b^	21.7 ± 3.0 ^b^	5.5 ± 0.6	6.6 ± 0.5 ^b^
	6	1.6 ± 0.2	2.8 ± 0.4	10.5 ± 2.0 ^b^	13.7 ± 2.2 ^b^	5.6 ± 1.0	7.5 ± 1.2 ^a^
	9	1.7 ± 0.2	2.4 ± 0.3	9.1 ± 2.9 ^b^	18.7 ± 4.7 ^ab^	6.0 ± 1.0	8.7 ± 1.0 ^b^

Different letters indicate statistically significant differences between stimuli (*p* < 0.05). Φ_PSII_, effective quantum yield of photochemical reactions of photosystem II; NPQ, non-photochemical fluorescence quenching.

**Table 2:** Duration and latency of the fast and long phases of photosynthetic inactivation for ΦPSII upon different local stimulations in detached wheat leaves treated with inhibitors.

Treatment	Stimulus	Duration of the Fast Phase of Φ_PSII_, min	Duration of the Long Phase of Φ_PSII_, min	Latency of the Long Phase of Φ_PSII_, min
Na_3_VO_4_	heating	2.2 ± 0.4	42.0 ± 9.5	9.3 ± 1.7 *
	burning	2.0 ± 0.4	47.3 ± 8.1 *	6.2 ± 1.3 *
	wounding	2.6 ± 0.6	57.6 ± 5.0 *	6.2 ± 1.1
LaCl_3_	heating	3.6 ± 1.0 *	38.1 ± 4.5	12.6 ± 2.6 *
	burning	2.8 ± 0.5 *	38.5 ± 9.5 *	9.1 ± 2.3 *
	wounding	3.9 ± 0.9 *	56.1 ± 3.7 *	5.6 ± 0.9
GdCl_3_	heating	2.6 ± 0.6	14.6 ± 4.5 *	4.5 ± 0.7
	burning	2.0 ± 0.4	7.2 ± 1.4 *	4.6 ± 0.7
	wounding	2.0 ± 0.4	10.2 ± 1.8	7.0 ± 1.6
DMTU	heating	1.3 ± 0.2	31.5 ± 5.1	9.2 ± 1.6 *
	burning	1.3 ± 0.2	13.8 ± 2.7	5.8 ± 0.8
	wounding	2.4 ± 0.8	2.5 ± 1.1 *	5.3 ± 1.7

* indicates data significantly different from untreated leaves (*p* < 0.05). Φ_PSII_, effective quantum yield of photochemical reactions of photosystem II.

## Data Availability

The original contributions presented in this study are included in the article/Supplementary Material. Further inquiries can be directed to the corresponding author.

## Supplementary Materials

The following supporting information can be downloaded at: https://www.mdpi.com/article/10.3390/ijms27010401/s1.

## Abbreviations

The following abbreviations are used in this manuscript:

SAA	Systemic acquired acclimation
VP	Variation potential
ROS	Reactive oxygen species
JAs	Jasmonates
Φ_PSII_	Effective quantum yield of photochemical reactions of photosystem II
NPQ	Non-photochemical fluorescence quenching
CWSI	Crop Water Stress Index
ABA	Abscisic acid
SA	Salicylic acid
JA	Jasmonic acid
JA-Ile	7-isojasmonoyl-L-isoleucine
DMTU	N,N′-dimethylthiourea
VDE	Violaxanthin de-epoxidase
PsaN	Subunits of photosystem 1
NADK2	NAD kinase 2
FDPase	Fructose-l,6-bisphosphatase
SBPase	Sedoheptulose-1,7-bisphosphatase
CP12	12 kDa chloroplast protein
GLRs	Glutamate receptor-like proteins
MscS	Mechanosensitive channel of small conductance
MSL10	MscS-like 10
LHCII	Light-harvesting complex II
TPC1	TWO PORE CHANNEL 1
TPK	TWO PORE POTASSIUM
elf18	Translation elongation factor Tu
flg22	Fragment of bacterial flagellin
