# Supplementary material for: Mechanisms of Induction of Stimulus-Specific Systemic Responses of Photosynthesis in Wheat Plants
Source: Int J Mol Sci. 2025 Dec 30;27(1):401. doi: 10.3390/ijms27010401 (PMC12786015; doi:10.3390/ijms27010401)
Supplement: Supplementary file 1 [file ijms-27-00401-s001.zip › ijms-4052534-supplementary.pdf]

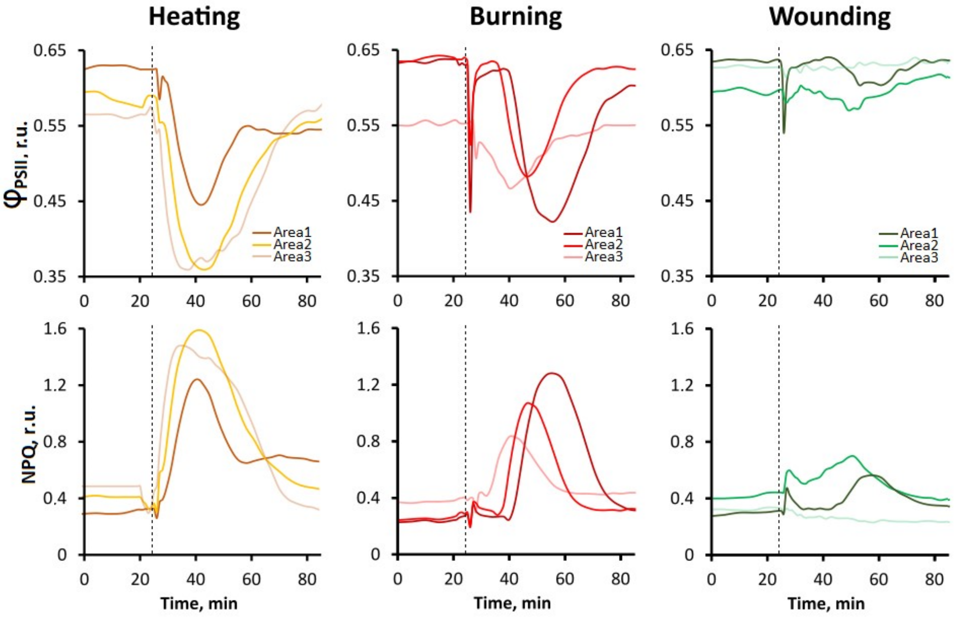

Figure S1. Representative recordings of systemic photosynthetic responses induced by local heating, burning or wounding in whole wheat plants. The dashed line indicates the moment of generation of the variation potential. Φ_{PSII}, effective quantum yield of photochemical reactions of photosystem II; NPQ, non-photochemical fluorescence quenching.

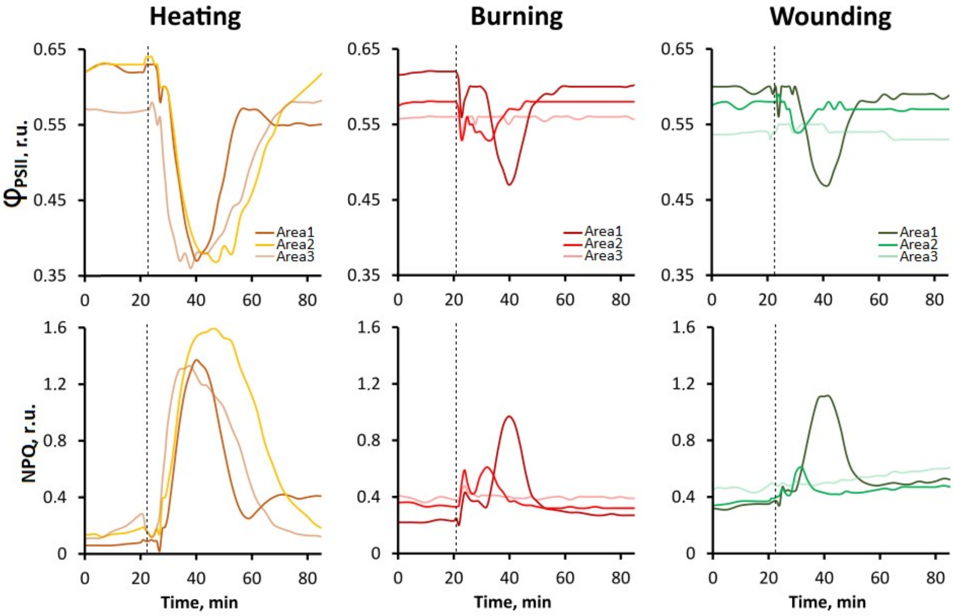

Figure S2. Repres entative recordings of systemic photosynthetic responses induced by local heating, burning or wounding in detached wheat leaves. The dashed line indicates the moment of generation of the variation potential. Φ_{PSII}, effective quantum yield of photochemical reactions of photosystem II; NPQ, non-photochemical fluorescence quenching.

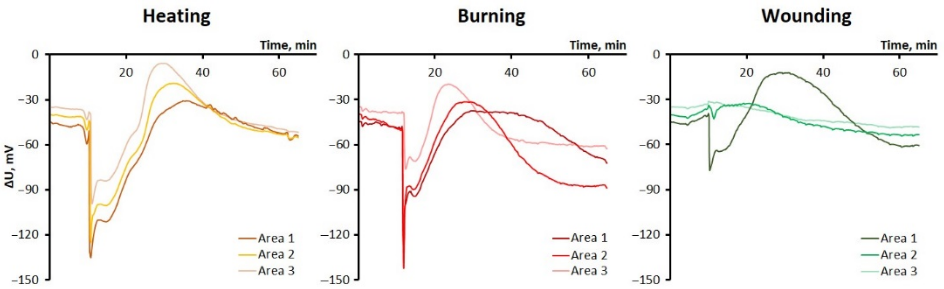

Figure S3. Representative recordings of variation potentials induced by local heating, burning or wounding in detached wheat leaves. ΔU, potential difference.

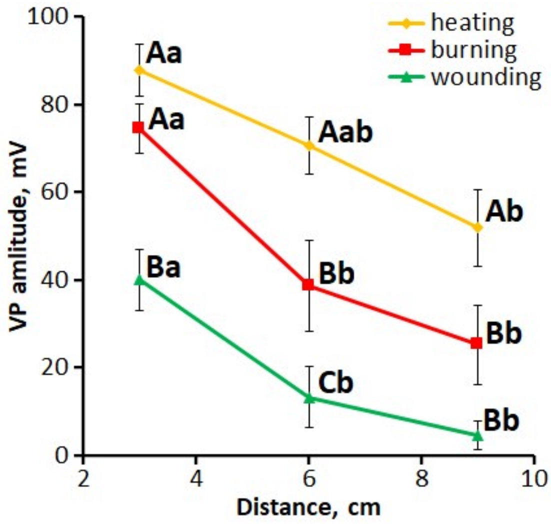

Figure S4. Dependence of the amplitudes of variation potential (VP) on the distance to the area of local stimulation in detached wheat leaves. Data are means ± SEM. Different uppercase letters indicate statistically significant differences between stimuli; different lowercase letters indicate statistically significant differences between distances within a single stimulus ($p < 0.05$).

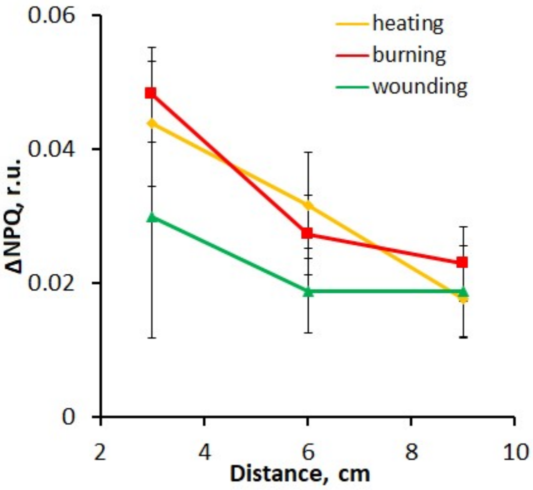

Figure S5. Dependence of the amplitudes of the initial photosynthetic activation on the distance to the area of local stimulation in detached wheat leaves. Data are represented as Mean ± SEM. NPQ, non-photochemical fluorescence quenching.

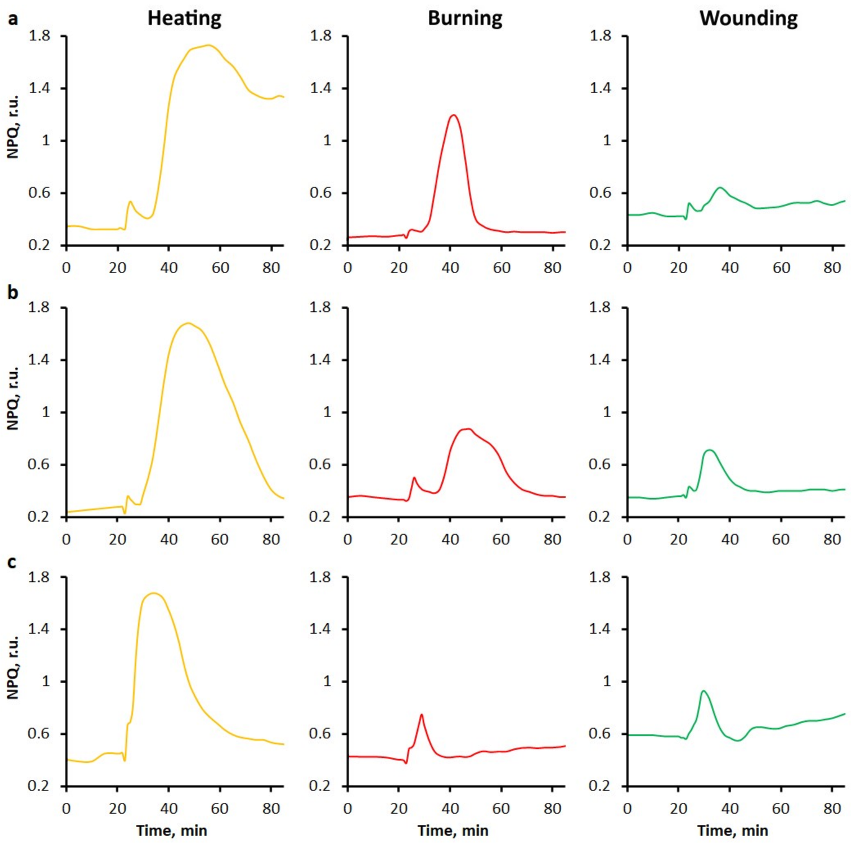

Figure S6. Representative recordings of systemic photosynthetic responses with small (a), medium (b) and large (c) extent of overlap of phases induced by local heating, burning or wounding in detached wheat leaves. The dashed line indicates the moment of generation of the variation potential. NPQ, non-photochemical fluorescence quenching.

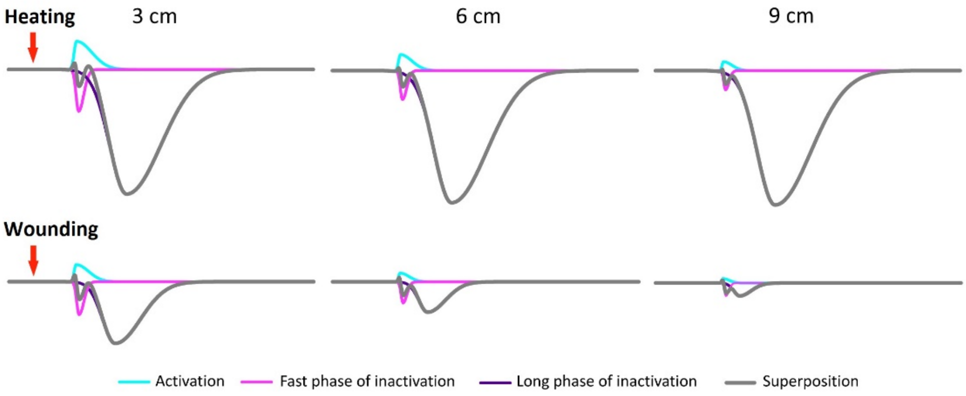

Figure S7. Hypothetical model for the formation of a multiphasic photosynthetic response caused by heating and wounding as a superposition of individual components induced by long-distance signals at different distances from the stimulation area. The components of the response are presented as curves corresponding to the initial activation (cyan), fast (magenta) and long (purple) phases of inactivation. Superposition of putative components represents changes in effective quantum yield of photochemical reactions of photosystem II.

Table S1. Slope of the fast and long phases of photosynthetic inactivation for ΦPSII and NPQ upon different local stimulations in detached wheat leaves.

Stimulus	Distance, cm	Slope of the fast phase, r.u./min		Slope of the long phase, r.u./min	
		Φ_{PSII}	NPQ	Φ_{PSII}	NPQ
heating	3	0.023±0.006	0.040±0.021	0.008±0.001	0.040±0.004
	6	0.027±0.007	0.084±0.019	0.013±0.002	0.066±0.006
	9	0.008±0.003	0.060±0.015	0.012±0.002	0.076±0.010
burning	3	0.041±0.008	0.052±0.012	0.012±0.002	0.052±0.007
	6	0.027±0.007	0.055±0.009	0.011±0.001	0.061±0.007
	9	0.015±0.004	0.036±0.008	0.009±0.002	0.088±0.033
wounding	3	0.018±0.004	0.036±0.006	0.007±0.001	0.038±0.005
	6	0.015±0.007	0.037±0.008	0.008±0.002	0.049±0.009
	9	0.018±0.010	0.026±0.008	0.006±0.002	0.043±0.010

Φ_{PSII}, effective quantum yield of photochemical reactions of photosystem II; NPQ, non-photochemical fluorescence quenching.
